# Distinct amyloid-dependent patterns of nigra dopamine depletion in Lewy body diseases

**DOI:** 10.3389/fnagi.2023.1196602

**Published:** 2023-08-08

**Authors:** Kyoungwon Baik, Jungho Cha, Mincheol Park, Younggun Lee, Seok Jong Chung, Han Soo Yoo, Young H. Sohn, Phil Hyu Lee

**Affiliations:** ^1^Department of Neurology, Yonsei University College of Medicine, Seoul, Republic of Korea; ^2^Nash Family Center for Advanced Circuit Therapeutics, Icahn School of Medicine at Mount Sinai, New York, NY, United States; ^3^Department of Neurology, Chung-Ang University College of Medicine and Graduate School of Medicine, Gwangmyeong Hospital, Gwangmyeong, Republic of Korea; ^4^Severance Biomedical Science Institute, Yonsei University College of Medicine, Seoul, Republic of Korea

**Keywords:** Parkinson's disease, dementia with Lewy bodies, Lewy body disease, florbetaben PET, dopamine transporter PET

## Abstract

**Introduction:**

Concomitant amyloid pathology is not uncommon and contributes to the clinical characteristics of Lewy body disease (LBD). We investigated the effect of amyloid on striatal^18^F-FP-CIT uptake patterns in LBD, including Parkinson's disease (PD) and dementia with Lewy bodies.

**Methods:**

We enrolled 125 patients with LBD who underwent^18^F-florbetaben positron emission tomography (PET) and^18^F-FP-CIT PET. Patients were divided into amyloid-positive and amyloid-negative groups. We investigated the effect of amyloid on striatal dopamine transporter (DAT) availability, depending on the type of LBD, using general linear models with interaction analysis after controlling for age, sex, education, deep white matter hyperintensity (WMH), periventricular WMH, and cognitive status.

**Results:**

There was a significant interaction effect between the disease group and the presence of amyloid on DAT availability in the anterior putamen, posterior putamen, caudate, and ventral striatum. In the presence of amyloid, only the PD group exhibited decreased DAT availability in the anterior and posterior putamen. In both groups, the presence of amyloid was not associated with DAT availability in the caudate and ventral striatum. The presence of amyloid was not directly related to the worse parkinsonian motor symptoms in both groups. However, there was a significant indirect effect of amyloid on parkinsonian motor symptoms, which was mediated by anterior and posterior putaminal DAT availability in the PD group alone.

**Discussion:**

This study demonstrates different amyloid-dependent or amyloid-independent^18^F-FP-CIT PET patterns in patients with LBD, suggesting distinctive interactions between α-synuclein and amyloid pathology based on the type of LBD.

## 1. Introduction

Lewy body disease (LBD) encompasses Parkinson's disease (PD), PD dementia (PDD), and dementia with Lewy bodies (DLBs). The LBDs share pathological hallmarks, Lewy bodies, and similar clinical characteristics, including rapid eye movement sleep behavior disorder (RBD), parkinsonism, fluctuating cognition, and visual hallucinations. The only distinction between PDD and DLB is the time relationship between the onset of dementia and motor symptoms (McKeith et al., 2017); however, cognitive impairment or prognosis and caregiver burden are more severe in DLB than in PDD (Walker et al., 2007; Yoon et al., 2014; Jellinger, 2018).

Along with Lewy bodies, concomitant Alzheimer's disease (AD) pathology is not uncommon and contributes to the clinical characteristics of LBD. Amyloid deposition was related to worse attention-executive and language functions in LBD patients (Biundo et al., 2021). In patients with DLB, a higher amyloid burden is associated with the diagnosis of dementia at a younger age, greater cognitive decline (Sarro et al., 2016), lower cognitive performance (Ferreira et al., 2020), decreased dopamine transporter (DAT) activity (Yoo et al., 2020), and more extensive gray matter atrophy (Sarro et al., 2016; Lee et al., 2018b). Similarly, compared to a lower amyloid burden, a higher amyloid burden is related to reduced cognitive performance (McMillan and Wolk, 2016), faster cognitive decline (Gomperts et al., 2013; Yarnall et al., 2014), shorter time-to-dementia onset (Compta et al., 2011), and increased postural instability and gait difficulty (Muller et al., 2013) in patients with PD.

Pathological and imaging studies have provided ample evidence that patients with DLB have a higher amyloid burden than those with PD (Petrou et al., 2012; Hepp et al., 2016). Additionally, the type and distribution of amyloid in DLB differ from those in PD. For example, the neuritic plaque score is higher in DLB than in PDD, and amyloid is more frequently observed in the entorhinal cortex, amygdala, and putamen in DLB than in PDD (Hepp et al., 2016). Another study showed that patients with DLB had frequent amyloid deposition in the midbrain, while patients with PD had less or scent amyloid deposition in the midbrain (Fujishiro et al., 2010; Sierra et al., 2016; Jellinger, 2023). Moreover, recent studies have reported that the different clinical and pathological features of LBD may be ascribed to the underlying differences in α-synuclein strains (Candelise et al., 2019; Van der Perren et al., 2020), which may also lead to different interactions with other proteins (Sengupta et al., 2020). One autopsy study showed different distribution patterns of α-synuclein pathology in the LBD spectrum depending on the concomitant AD (Toledo et al., 2016). Although the biological effects of mixed pathologies or interactions between α-synuclein and other toxic proteins on neuronal degeneration have been suggested (Sengupta et al., 2020), little is known about how different α-synuclein strains would interact with other proteins.

How does concomitant amyloid accumulation affect the nigrostriatal dopaminergic system in the LBD spectrum and their relationship with parkinsonian motor symptoms is elusive? In this study, we hypothesized that amyloid pathology may interact in a different manner with the nigrostriatal dopaminergic system in DLB and PD; therefore, the patterns of nigrostriatal DAT availability would differ. To test this hypothesis, we performed an interaction analysis in patients with DLB and PD who had undergone ^18^F-florbetaben positron emission tomography (^18^F-FBB PET) to examine specific ^18^F-N-(3-fluoropropyl)-2β-carbon ethoxy-3β-(4-iodophenyl) nortropane (^18^F-FP-CIT) PET uptake, which interacts with amyloid depending on the type of LBD.

## 2. Materials and methods

### 2.1. Participants

We reviewed the medical records of 125 patients with PD or DLB who visited the movement and dementia clinics of Yonsei University Severance Hospital between July 2015 and December 2020. PD was diagnosed according to the United Kingdom Parkinson's Disease Brain Bank diagnostic criteria. PD with mild cognitive impairment (MCI) and PDD were diagnosed according to the level II PD-MCI criteria (Litvan et al., 2011) and the clinical criteria of probable PDD, respectively (Dubois et al., 2007). DLB was diagnosed using the 2017 revised criteria (McKeith et al., 2017); all patients satisfied the probable DLB criteria. All patients with MCI due to DLB met the probable DLB criteria, except for the presence of dementia. All patients in both groups underwent ^18^F-FP-CIT PET and were confirmed to have dopaminergic depletion. We excluded patients with severe white matter hyperintensities (WMHs), multiple lacunes in the basal ganglia, or hydrocephalus on magnetic resonance imaging, as well as patients with other neurologic, psychiatric, or metabolic illnesses.

All patients underwent a neurologic examination. The severity of parkinsonism was assessed according to the Unified Parkinson's Disease Rating Scale (UPDRS) Part III score in the off-state. The Korean version of the Mini-Mental State Examination (K-MMSE) and the clinical dementia rating scale sum of boxes (CDR-SOB) were used to evaluate general cognition and staging dementia severity, respectively. Clinical features of LBD, including RBD, visual hallucinations, and cognitive fluctuation, were evaluated using semi-structured questionnaires, which were administered by caregivers. All patients underwent ^18^F-FBB PET. This study was approved by the Yonsei University Severance Hospital Institutional Review Board (No. 4-2016-0210), and the need for informed consent was waived because of the retrospective design of the study.

### 2.2. Acquisition and processing of MR data

In total, 71 participants were scanned using a Philips 3.0 T MR scanner (Philips Achieva; Philips Medical Systems, Best, The Netherlands) with a SENSE head coil (SENSE factor = 2). T1-weighted magnetic resonance imaging (MRI) data were obtained using a 3D T1-TFE sequence with the following parameters: 224 × 224 axial acquisition matrix; 256 × 256 reconstructed matrix with 170 slices; voxel size, 0.859 × 0.859 × 1 mm; field of view, 220 mm; echo time, 4.6 ms; repetition time, 9.8 ms; and flip angle, 8°. With the baseline image without weighting, diffusion-weighted MRI data were acquired from 32 diffusion sampling directions using a single-shot echo-planar acquisition with the following parameters: 128 × 128 acquisition matrix with 70 slices; voxel size, 1.75 × 1.75 × 2 mm; field of view, 220 mm; b-factor, 600 s/mm^2^; echo time, 70 ms; repetition time, 7.663 sec; and flip angle, 90°. Conventional two-dimensional fluid-attenuated inversion recovery images were obtained to evaluate white matter hyperintensities.

### 2.3. Measurement of regional white matter hyperintensities

A visual rating scale for WMH was modified from the Fazekas scale. Periventricular WMH (PWMH) areas were classified as P1 (cap and band < 5 mm), P2 (5 mm ≤ cap or band < 10 mm), and P3 (10 mm ≤ cap or band); deep WMH (DWMH) areas were classified as D1 (maximum diameter of deep white matter lesion < 10 mm), D2 (10 mm ≤ lesion < 25 mm), and D3 (≥ 25 mm).

### 2.4. Acquisition and interpretation of ^18^F-FBB PET scans

Detailed methods for ^18^F-FBB PET acquisition have been described in a previous study (Lee et al., 2018b). Quantitative PET image analysis was performed using a PET template (Kim and Jk, 2020). ^18^F-FBB PET images were then co-registered to the PET template using non-linear registration in advanced normalization tools to obtain the affine transformation matrix and deformation field. The inverse transformation was performed to warp the parcellation mask from the standard space of the PET template to the native space of each PET image. The participant-specific mask image comprised 68 cortical and 10 subcortical parcellated regions of the Desikan–Killiany Atlas (Klein and Tourville, 2012). Standardized uptake value ratios (SUVRs) were calculated according to the ADNI Florbetaben PET processing performed by UC Berkley (Sabri et al., 2015). Subregional SUVRs were calculated for the four predefined cortical regions of interest (ROIs) (frontal, lateral temporal, lateral parietal, and anterior/posterior cingulate) by overlaying the participant-specific composite masks. The whole cerebellum was used as the reference region. In addition, global SUVRs were established for each participant by volume-weighted averaging across four cortical regions and dividing this by the reference region. Patients who had composite Aβ retention above the threshold for Aβ positivity (global SUVRs > 1.20) were regarded as β-amyloid-positive (Martersteck, 2020) (see also https://adni.bitbucket.io/reference/docs/UCBERKELEYFBB/UCBerkeley_FBB_Methods_04.11.19.pdf).

### 2.5. Acquisition and quantitative analyses of ^18^F-FP-CIT PET images

The ^18^F-FP-CIT PET scans were acquired using a GE PET-CT DSTe scanner (GE Discovery STE; GE Healthcare; Milwaukee, WI, USA), which obtains images with a three-dimensional resolution of 2.3 mm full width at half maximum. All subjects were instructed to fast for ≥6 h before the PET scan, and 5mCi (185 MBq) of ^18^F-FP-CIT was intravenously administered to the subjects. After 90-min post-injection, images were acquired for 20 min in the three-dimensional mode.

Image processing was conducted using the SPM12 (Statistical Parametric Mapping 12, Wellcome Department of Imaging Neuroscience, Institute of Neurology, London, England) software package. For each patient, the reconstructed PET image was spatially normalized to the study-specific ^18^FP-CIT PET template created by a subset of two groups, including 44 patients with DLB and 27 patients with PD. To create the study-specific ^18^FP-CIT PET template, individual PET images were co-registered with the T1-weighted anatomical image and then normalized to the MNI152 template using the normalization parameters defined from the T1-weighted anatomical image. Then, the intensity was normalized by the uptake value of the occipital volume-of-interests (VOIs). The occipital VOI, including the calcarine fissure and surrounding cortex (V1), was selected from the AAL atlas. Finally, the processed 71 patients' PET images were averaged to generate a study-specific ^18^FP-CIT PET template. The striatum was divided into the caudate, anterior putamen, posterior putamen, and ventral striatum. The specific-to-non-specific binding ratio or DAT availability of each striatal subregion was defined as follows: (mean standardized uptake value of the striatal subregion VOIs—mean standardized uptake value of the occipital VOI)/(mean standardized uptake value of the occipital VOI). In addition, we further investigated two characteristic DAT patterns, which are the anterior–posterior gradient and right-left asymmetry. The inter-subregional ratio (ISR) was calculated by dividing the DAT availability of the anterior putamen by that of the posterior putamen. The ISR represents the anterior–posterior gradient of DAT uptake within the striatum. The asymmetricity ratio (AR) was calculated by dividing the DAT availability of the less affected posterior putamen by that of the more affected posterior putamen.

### 2.6. Statistical analysis

Statistical analyses were performed using the Statistical Package for the Social Sciences version 26.0 (IBM Corp., Armonk, NY). The independent *t*-test and χ2 test were performed to compare demographics and clinical features across disease groups.

Initially, we evaluated the differences in subregional DAT availability, ISR, and AR between the PD and DLB groups using general linear models (GLMs) after controlling for age, sex, DWMH, PWMH, and cognitive status. Cognitive status was regarded as a binary variable; 0 for patients without dementia and 1 for patients with dementia. We used cognitive status as a covariate since there was a significant difference in the proportion of patients with dementia between the two disease groups. Next, we evaluated the interaction effects of the disease group and the presence of amyloid on subregional DAT availability, ISR, AR, and the UPDRS Part III score using GLM after controlling for age, sex, DWMH, PWMH, and cognitive status. If there was a significant interaction effect between the disease group and the presence of amyloid, we investigated the effect of the presence of amyloid on subregional DAT availability or UPDRS Part III score in the DLB and PD groups separately. If the interaction term was not significant, GLM analyses were performed for all participants, regardless of group. In addition, the disease group and the presence of amyloid were entered as predictors. We applied the false discovery rate (FDR) method to correct multiple comparisons and an interaction term with Q (FDR-corrected *P*-value) < 0.2 was considered to be significant (Selvin, 2004).

We further investigated whether amyloid affects parkinsonian motor symptoms through the nigra dopaminergic pathway. Mediation analyses were used to evaluate whether DAT availability of the anterior or posterior putamen mediates associations between amyloid and UPDRS Part III scores after controlling for age, sex, DWMH, and PWMH in each disease group. Since dopamine depletion in the putamen is a core pathological feature and best reflects disease severity in patients with PD (Fearnley and Lees, 1991), we chose the anterior putamen and posterior putamen for further analysis. We used the PROCESS macro in SPSS with 5,000 bootstrapped samples for mediation analysis. A confidence interval (CI) that does not contain zero indicates a significant mediation effect.

## 3. Results

### 3.1. Demographics and clinical characteristics

A total of 125 patients with LBD (82 patients with DLB and 43 patients with PD) were included in the study. Patient demographics are presented in Table 1. There were no significant differences in age, sex, education level, UPDRS Part III score, DWMH, and PWMH between the groups. The MMSE score was lower in the DLB group than in the PD group (*P* < 0.001). In addition, the CDR-SOB score and proportion of patients with dementia were higher in the DLB group than in the PD group (*P* < 0.001). Patients with DLB experienced fluctuations and hallucinations more frequently than those with PD (*P* < 0.001). The proportion of patients with RBD was similar between the groups. Amyloid positivity was higher in the DLB group than in the PD group (53.7 vs. 20.9%; *P* < 0.001).

**Table 1:** Demographics and clinical characteristics.

	**DLB**	**PD**	***P*-value**
*N*	82	43	
Age	76.5 (6.1)	76.7 (6.3)	0.838
Sex, male (%)	40 (48.8)	27 (62.8)	0.136
Education	10.6 (5.0)	9.8 (5.2)	0.396
K-MMSE	20.2 (5.3)	24.1 (4.0)	< 0.001
CDR-SOB	5.0 (3.1)	2.1 (1.8)	< 0.001
UPDRS part III	24.3 (10.5)	22.2 (8.5)	0.295
DWMH	1.3 (0.8)	1.3 (0.9)	0.921
PWMH	1.7 (0.8)	1.7 (0.9)	0.996
RBD, *N* (%)	42 (51.2)	24 (55.8)	0.625
Visual hallucination, *N* (%)	37 (28.2)	6 (14.0)	< 0.001
Fluctuation, *N* (%)	35 (42.7)	2 (4.7)	< 0.001
Dementia, *N* (%)	70 (85.4)	14 (32.6)	< 0.001
Amyloid positivity, *N* (%)	44 (53.7)	9 (20.9)	< 0.001

Data are expressed in mean (standard deviation) or number (percentage). Group comparisons were performed using chi-square tests or analyses of variance as appropriate.

CDR-SOB, clinical dementia rating scale sum of boxes; DLB, dementia with Lewy bodies; DWMH, deep WMH; K-MMSE, Korean version of mini-mental state examination; PD, Parkinson's disease; PWMH, periventricular WMH; RBD, REM sleep behavior disorder; UPDRS, unified Parkinson's disease rating scale; WMH, white matter hyperintensity.

### 3.2. DAT availability in patients with PD and DLB

The subregional DAT availability, ISR, and AR in the patients with PD and DLB are shown in Table 2. There was no significant difference between DAT availability in the anterior putamen, caudate, and ventral striatum. DAT availability in the posterior putamen was higher in patients with DLB than those with PD (3.51 ± 1.12 vs. 3.10 ± 1.13; *P* = 0.037), but this relationship was not significant after correction for the multiple comparison (*Q* = 0.111). In addition, patients with both PD and DLB had similar patterns of symmetry in the bilateral posterior putamen. The ISR in the putamen was higher in the PD group than in the DLB group (1.14 ± 0.22 vs. 1.03 ± 0.16; *P* = 0.021), but this relationship was not significant after correction for the multiple comparison (*Q* = 0.111).

**Table 2:** Subregional DAT availability in each disease group.

	**DLB**	**PD**	***P*-value**	***Q*-value**
Anterior putamen	3.55 (1.07)	3.42 (1.02)	0.242	0.484
Posterior putamen	3.51 (1.12)	3.10 (1.13)	0.037	0.111
Caudate	2.25 (0.82)	2.28 (0.85)	0.485	0.723
Ventral striatum	2.62 (0.73)	2.77 (0.88)	0.794	0.794
AR	1.12 (0.15)	1.15 (0.20)	0.765	0.794
ISR	1.03 (0.16)	1.14 (0.22)	0.021	0.111

Data are expressed in mean (standard deviation) or number (percentage). Group comparisons are performed using the general linear model after controlling for age, sex, cognitive status, DWMH, and PWMH. *Q*-value is the FDR-corrected *P*-value to correct multiple comparison for four regression analyses.

AR, asymmetricity ratio; DLB, dementia with Lewy bodies; DWMH, deep WMH; ISR, inter-subregional ratio; PD, Parkinson's disease; PWMH, periventricular WMH; WMH, white matter hyperintensity.

### 3.3. Effect of amyloid on the subregional DAT availability

**Figure 1:**
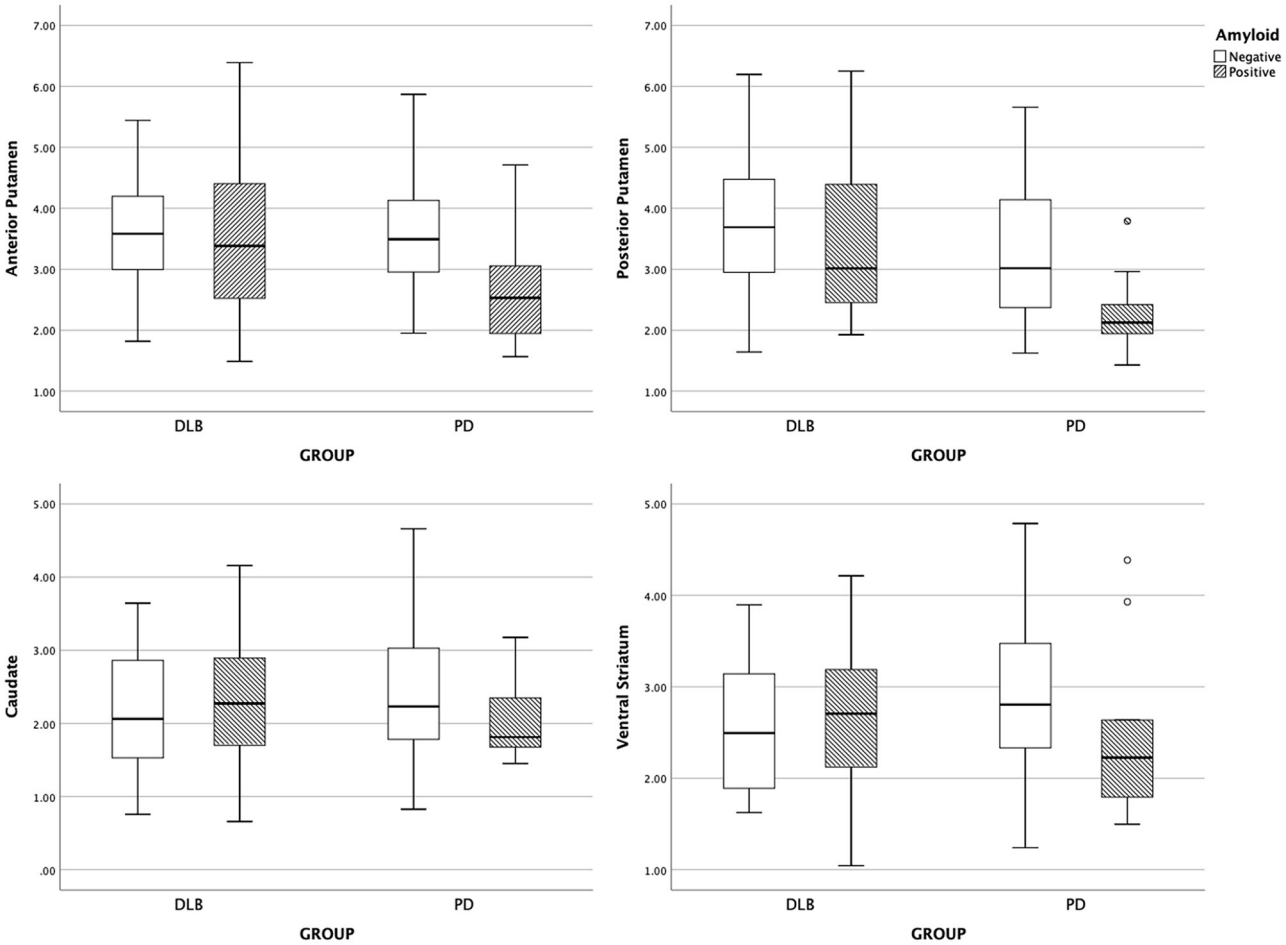
Boxplot of subregional DAT uptake. DLB, dementia with Lewy bodies; PD, Parkinson's disease.

The subregional DAT availability according to amyloid positivity is shown for each group in Figure 1. The results of the interaction analysis on DAT availability are shown in Table 3. The interaction effect between the disease group and the presence of amyloid was significant for DAT availability in the anterior putamen, posterior putamen, caudate, and ventral striatum (*Q* = 0.093) (Table 3). Thus, further analyses were performed within each group.

**Table 3:** Interaction analysis.

	***P*-value**	***Q*-value**
Anterior putamen	0.018	0.093
Posterior putamen	0.037	0.093
Caudate	0.062	0.093
Ventral striatum	0.054	0.093
AR	0.792	0.899
ISR	0.899	0.899

Data are the significance of the interaction effects of the disease group and the presence of amyloid on the subregional DAT availability using GLM after controlling for age, sex, DWMH, PWMH, and cognitive status.

*Q*-value is the FDR-corrected *P*-value to correct multiple comparisons for four regression analyses.

AR, asymmetricity ratio; DWMH, deep WMH; FDR, false discovery rate; GLM, general linear model; ISR, inter-subregional ratio; PWMH, periventricular WMH; WMH, white matter hyperintensity.

With the presence of amyloid, the anterior putaminal DAT availability further decreased from 3.61 to 2.69 (*B* = −1.12, *Q* = 0.016), and the posterior putaminal DAT availability decreased from 3.32 to 2.28 (*B* = −1.20, *Q* = 0.016) in the PD group. However, the presence of amyloid had no effect on DAT availability in the ventral striatum (*Q* = 0.167) and caudate (*Q* = 0.358) in the PD group. In patients with DLB, the presence of amyloid exerted no effect on the DAT availability in striatal subregions, including the anterior putamen (*Q* = 0.705), posterior putamen (*Q* = 0.520), caudate (*Q* = 0.520), or ventral striatum (*Q* = 0.588) (Table 4).

**Table 4:** Effect of amyloid on the subregional DAT uptake in each group.

	**DLB**		**Amyloid effect**			**PD**		**Amyloid effect**		
	**Amyloid (–)**	**Amyloid (**+**)**	**B (SE)**	***P*** **-value**	***Q*** **-value**	**Amyloid (–)**	**Amyloid (**+**)**	**B (SE)**	***P*** **-value**	***Q*** **-value**
Anterior putamen	3.57 (0.94)	3.53 (1.18)	−0.09 (0.23)	0.705	0.705	3.61 (0.96)	2.69 (0.96)	−1.12 (0.39)	0.007	0.016
Posterior putamen	3.64 (1.08)	3.40 (1.16)	−0.29 (0.23)	0.210	0.520	3.32 (1.12)	2.28 (0.73)	−1.20 (0.43)	0.008	0.016
Caudate	2.16 (0.81)	2.33 (0.84)	0.19 (0.17)	0.260	0.520	2.35 (0.90)	2.01 (0.57)	−0.30 (0.32)	0.358	0.358
Ventral striatum	2.55 (0.67)	2.69 (0.78)	0.12 (0.16)	0.441	0.588	2.85 (0.84)	2.50 (1.02)	−0.51 (0.33)	0.125	0.167

The results are from the GLM analysis for subregional DAT availability which showed a significant interaction effect between the group and the presence of amyloid. GLM analysis was performed in each group. The presence of amyloid was entered as predictor after controlling for age, sex, cognitive status, DWMH, and PWMH.

*Q*-value is the FDR-corrected *P*-value to correct multiple comparison for four regression analyses.

B, unstandardized coefficient; DLB, dementia with Lewy bodies; DWMH, deep WMH; FDR, false discovery rate; GLM, general linear model; PD, Parkinson's disease; PWMH, periventricular WMH; WMH, white matter hyperintensity.

In addition, since the interaction effect between the disease group and the presence of amyloid was not significant on the AR and ISR (*Q* = 0.899), further analyses were performed within both groups (Table 3). With the presence of amyloid, the AR increased from 1.08 ± 0.09 to 1.15 ± 0.17 in the DLB group and 1.13 ± 0.19 to 1.23 ± 0.23 in the PD group, showing no between-group difference. The presence of amyloid exerted no effect on the ISR in both the PD and DLB groups. However, the ISR was higher in the PD group than in the DLB group (*Q* = 0.016) (Table 5).

**Table 5:** Effect of amyloid and group on the AR and ISR.

	**DLB**		**PD**		**Group effect**			**Amyloid effect**		
	**Amyloid (–)**	**Amyloid (**+**)**	**Amyloid (–)**	**Amyloid (**+**)**	**B (SE)**	***P*** **-value**	***Q*** **-value**	**B (SE)**	***P*** **-value**	***Q*** **-value**
AR	1.08 (0.09)	1.15 (0.17)	1.13 (0.19)	1.23 (0.23)	−0.039 (0.038)	0.301	0.301	0.083 (0.031)	0.009	0.018
ISR	1.00 (0.13)	1.05 (0.18)	1.13 (0.22)	1.19 (0.22)	−0.112 (0.041)	0.008	0.016	0.054 (0.034)	0.118	0.118

The results are from the GLM analysis for AR and ISR which showed no significant interaction effect between the group and the presence of amyloid. GLM analysis was performed in both groups. The presence of amyloid and group was entered as predictors after controlling for age, sex, cognitive status, DWMH, and PWMH.

*Q*-value is the FDR-corrected *P*-value to correct multiple comparison for two regression analyses.

B, unstandardized coefficient; AR, asymmetricity ratio; DLB, dementia with Lewy bodies; DWMH, deep WMH; GLM, general linear model; ISR, inter-subregional ratio; PD, Parkinson's disease; PWMH, periventricular WMH; WMH, white matter hyperintensity.

### 3.4. Effect of amyloid on the UPDRS Part III score

**Figure 2:**
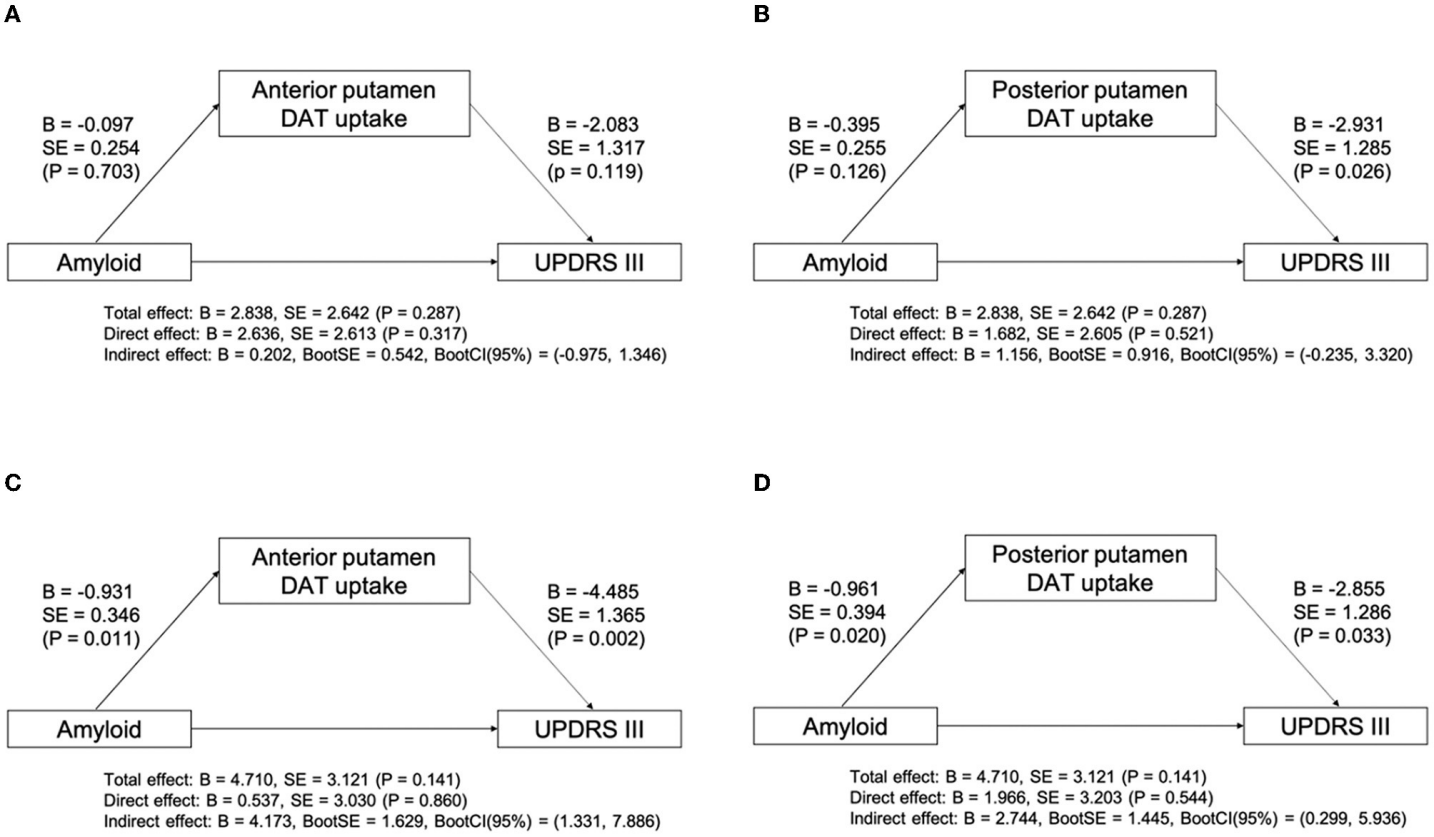
Mediation analysis between amyloid, subregional DAT uptake, and UPDRS Part III score after controlling for age, sex, DWMH, and PWMH in DLB **(A, B)** and PD **(C, D)** groups. B, unstandardized coefficient; BootCI, bootstrap confidence interval; BootSE, bootstrap SE; DWMH, deep WMH; DLB, dementia with Lewy bodies; P, *P*-value; PD, Parkinson's disease; PWMH, periventricular WMH; SE, standard error; UPDRS, unified Parkinson's disease rating scale; WMH, white matter hyperintensity.

We evaluated the relationship between the presence of amyloid and parkinsonian motor symptoms in patients with LBD. There was no significant interaction effect between the disease group and the presence of amyloid on parkinsonian motor symptoms (*P* = 0.291). Within both disease groups, there was no significant effect of group (*P* = 0.310), or amyloid (*P* = 0.081). We further evaluated the indirect effect of amyloid on the UPDRS Part III score using putaminal DAT availability as a mediator in each disease group. The mediation analysis revealed that there was no significant mediation effect of DAT availability in the anterior putamen or posterior putamen on the UPDRS Part III score in the DLB group (Figures 2A, B). However, the indirect effect of amyloid on the UPDRS Part III score through the mediation of DAT availability in the anterior putamen (indirect effect, *B* = 4.713; 95% CI, 1.331–7.886; Figure 2C) and posterior putamen (indirect effect, *B* = 2.744; 95% CI, 0.299–5.936; Figure 2D) was significant in the PD group.

## 4. Discussion

In this study, we found distinct amyloid-dependent or amyloid-independent ^18^F-FP-CIT PET patterns in patients with LBD. First, the presence of amyloid had a detrimental effect on the DAT availability in the anterior and posterior putamen in the PD group only. Second, the presence of amyloid was not associated with DAT availability in any striatal subregion in the DLB group. Third, the presence of amyloid was related to the increased asymmetricity of posterior putaminal DAT availability in both PD and DLB groups. Finally, additional amyloid pathology was not directly related to the parkinsonian motor symptom, but indirectly related to the increased UPDRS Part III score, which was mediated by DAT availability in the anterior and posterior putamen in the PD group only. These data suggest that the interaction between amyloid pathology and the nigrostriatal dopaminergic system is different between DLB and PD groups.

Although the location and mechanism of the interactions between toxic protein pathologies are yet to be elucidated in neurodegenerative diseases, several clinicopathological studies have provided evidence of a close intergroup association between toxic proteins. In patients with LBD including PD or DLB, a pathological study reported that the deposition of amyloid detected by [^11^C] Pittsburgh compound B had a significant correlation with Lewy body score and the severity of the stage of neurofibrillary tangle (Colom-Cadena et al., 2013; Shirvan et al., 2019). Furthermore, the presence of amyloid was associated with greater α-synuclein accumulation in LBD (Miller et al., 2022). In addition, Lewy body counts were significantly correlated with senile plaque counts and, to a lesser extent, neurofibrillary tangles in patients with PDD (Apaydin et al., 2002). Functional imaging studies using multiple radiotracers reported similar results that the burden of amyloid and tau proteins was closely correlated with each other in patients with DLB (Lee et al., 2018a). Importantly, the pattern of tau accumulation in DLB patients was distinctive compared to patients with AD.

Interestingly, we found that the presence of amyloid pathology in LBD had a distinct effect on the patterns of DAT availability depending on the specific LBD disease. Considering that subregional DAT availability in the striatum corresponds to dopaminergic neuronal projections from the substantia nigra (SN) (Fearnley and Lees, 1991), striatal DAT patterns in this study may represent region-specific dopaminergic neuronal loss in the SN. This study showed that the detrimental effect of amyloid on the lateral tier of nigra dopaminergic neurons that project to the putamen was dependent on the specific disease type of LBD. This means that the presence of amyloid led to significantly decreased DAT availability in the putamen, respective of the disease groups. In contrast, amyloid-independent DAT availability in the caudate and striatum was not specific to the type of LBD, such that the presence of amyloid had no significant effects on dopaminergic neurons outside the lateral portion of the SN in both groups. In addition, the detrimental effect of the presence of amyloid on the AR between the bilateral posterior putamen was significant in the PD group. This suggests that concomitant amyloid pathology influences more on the more affected side of the substantia nigra and that the interaction between amyloid and α-synuclein could have a synergistic effect rather than an additive effect. However, the presence of amyloid was associated with increased AR, without having a significant effect on the putaminal DAT availability in the DLB group. This suggests that the interaction between amyloid and α-synuclein in DLB patients may be present, at least on the more affected side of the substantia nigra, but overall, the interaction is less prominent than that in the PD group. These results may provide indirect evidence for the region- and disease-dependent effects of amyloid on the nigra dopaminergic neurons.

Recently, accumulating evidence has suggested that various clinicopathological traits in α-synucleinopathies may be ascribed to different α-synuclein strains (Shahnawaz et al., 2020; Holec and Woerman, 2021; Ayers et al., 2022). In terms of α-synuclein characteristics in patients with DLB, toxicity to the dopaminergic neurons in the SN appears to be less potent compared to α-synuclein in PD (Van der Perren et al., 2020). Moreover, Toledo et al. showed that demented subjects with AD and Lewy body-related pathology (Dem-AD-LB, which could partially overlap DLB with an amyloid group in our study) had less reduced substantia nigra neurons than PD with AD or PD without AD, and DLB without AD. Dem-AD-LB patients were less likely to show involvement in Lewy body-related pathology in the brainstem (Toledo et al., 2016). Therefore, disease-specific characteristics of α-synuclein strains would explain the more detrimental effects of concomitant amyloid on the putaminal DAT availability observed in the PD group compared to the DLB group. This result implies that the α-synuclein strain interacts differently with β-amyloid, depending on the specific type of α-synucleinopathies, as well as on specific regions of nigra dopaminergic neurons. Further clinicopathological data are needed to uncover the relationship between α-synuclein strains and other toxic proteins.

This study also demonstrated that additional amyloid pathology was indirectly related to the increased UPDRS Part III score. In addition, the detrimental effect of amyloid on parkinsonian motor symptoms in patients with LBD was disease-specific. The indirect effect of amyloid was present in the PD group only. In terms of neuroanatomical correlates of parkinsonian motor symptoms, it has been postulated that parkinsonism in patients with PD is based on neuronal loss in the SN, whereas parkinsonism in DLB is attributed to widespread anatomical structures beyond the SN (Postuma et al., 2012). Given that the different neural correlates of parkinsonism exist in LBD, it is speculated that the amyloid pathology may exert effects differently in the distinct anatomical area responsible for parkinsonism, depending on the type of LBD. The mediation analysis revealed that the effect of amyloid on motor deficits was mediated by DAT availability in the anterior and posterior putamen in the PD group. Considering that the effect of amyloid on DAT availability in the anterior and posterior putamen was LBD type-specific, additional neurotoxic effects of amyloid on nigra dopaminergic neurons projecting into the putamen that exhibits a disease-specific pattern may result in additive motor deficits in the PD group. To date, the location and mechanisms of the interaction between amyloid and synuclein pathologies remain to be elucidated. In our study, the presence of amyloid was associated with putaminal dopaminergic depletion, further correlating with motor dysfunction. Therefore, among striatal subregions in patients with PD, the putamen might be the primary region where the interaction between amyloid and synuclein pathologies occurs. This result may provide a clue for clinical relevance regarding the interaction of mixed pathologies in neurodegenerative diseases.

In terms of spatial or asymmetric patterns, the AR and ISR did not differ between the DLB and PD groups. Thus, there was no difference between the two groups in the asymmetry or anterior–posterior gradient of substantia nigra degeneration evaluated by DAT imaging. However, when considering the presence of amyloid, the anterior–posterior gradient of DAT availability in the putamen was more prominent in the PD group relative to the DLB group. There has been controversy over differences in patterns of striatal DAT availability between DLB and PD patients. The patterns of striatal DAT availability were compatible between two groups (Colloby et al., 2004; Marquie et al., 2014; Gomperts et al., 2016; Pilotto et al., 2019), whereas others reported lower caudate DAT availability in DLB patients and greater asymmetricity or antero-posterior gradient of DAT availability in PD patients (Ransmayr et al., 2001; O'Brien et al., 2004; Walker et al., 2004). Our results imply that the presence of amyloid may influence the spatial or asymmetric distribution of DAT availability in the PD and DLD groups, and thus, the prevalence of amyloid positivity may contribute to the different results in previous studies.

Our study has some limitations. First, the diagnosis of LBD was not pathologically confirmed; thus, an alternative diagnosis of DLB may be possible. However, all DLB patients in this study met the probable DLB criteria from the 2017 revised criteria for DLB. In addition, we used additional supportive imaging biomarkers to diagnose patients more accurately with DLB using ^18^F-FP-CIT PET. Second, there was a significant difference in the proportion of patients with dementia between the two groups. Although we tried to overcome this difference by using cognitive status as covariates, the results should be interpreted with caution. Third, we used a dichotomized approach to amyloid rather than global SUVR as a continuous variable. Traditionally, quantitative cutoffs for amyloid PET positivity have been defined to discriminate AD patients from non-AD patients. However, there have been some studies dealing with the subthreshold amyloid accumulation which has clinical significance for a worse prognosis (Bischof and Jacobs, 2019). Therefore, we performed additional analysis with global SUVR as a continuous variable. However, there was no significant interaction effect or independent effect of amyloid when we treated the amyloid uptake as a continuous variable (Supplementary Table 1). This might suggest that a substantial accumulation of amyloid is needed to have significant interaction with PD. Finally, the patients in our study had a relatively older onset age. Older onset age could affect the prevalence of amyloid positivity or be associated with aggravated nigra neuronal cell death. However, there was no difference in the onset age between the two groups (74.07 ± 6.47 in the DLB group and 73.81 ± 6.56 in the PD group).

In conclusion, DLB and PD interact with amyloid pathology differently with regard to nigrostriatal dopaminergic dysfunction, which could provide indirect evidence of the presence of different α-synuclein strains in synucleinopathies and their distinct interaction with amyloid pathology.

## Data availability statement

The data and code used in this work are available from the corresponding author upon reasonable request.

## Ethics statement

The studies involving human participants were reviewed and approved by Yonsei University Severance Hospital Institutional Review Board. Written informed consent for participation was not required for this study in accordance with the national legislation and the institutional requirements.

## Author contributions

KB, JC, and PL contributed to the conception and design of the manuscript. KB, SC, HY, and YS organized the database. KB, MP, YL, and JC performed the statistical analysis. KB wrote the first draft of the manuscript. JC, MP, and PL wrote sections of the manuscript. All authors contributed to the manuscript revision, read, and approved the submitted version.
