# Supplementary material for: Distinct amyloid-dependent patterns of nigra dopamine depletion in Lewy body diseases
Source: Front Aging Neurosci. 2023 Aug 8;15:1196602. doi: 10.3389/fnagi.2023.1196602 (PMC10442581; doi:10.3389/fnagi.2023.1196602)
Supplement: Supplementary file 1 [file Table_1.docx]

**Supplementary table 1. Interaction analysis**

|  | P value | Q value |
| --- | --- | --- |
| Anterior putamen | 0.420 | 0.840 |
| Posterior putamen | 0.381 | 0.840 |
| Caudate | 0.075 | 0.450 |
| Ventral striatum | 0.668 | 0.989 |
| AR | 0.989 | 0.989 |
| ISR | 0.845 | 0.989 |

Data are significance of interaction effects of the disease group and global amyloid SUVRs on the subregional DAT availability using GLM after controlling for age, sex, DWMH, PWMH and cognitive status.

Q value is FDR-corrected P value to correct multiple comparison for 4 regression analyses.

Abbreviations: AR, Asymmetricity ratio; DWMH, deep WMH; FDR, false discovery rate; GLM, general linear model; ISR, Intersubregional ratio; PWMH, Periventricular WMH; SUVRs, Standardized uptake value ratios; WMH, White matter hyperintensity
